# Effects of Flurochloridone Application on Rhizosphere Soil Fungal Community and Composition in Potato Growing Areas of the Qinghai-Tibet Plateau

**DOI:** 10.3390/jof7060420

**Published:** 2021-05-27

**Authors:** Wei Li, Shuo Shen, Hongyu Chen, Yang Zhang, Lei Deng, Yujiao Liu, Zhouping Shangguan

**Affiliations:** 1 State Key Laboratory of Soil Erosion and Dryland Farming on the Loess Plateau, Northwest A&F University, Xiangyang 712100, China; Liweilwbabylw@qhu.edu.cn (W.L.); leideng@ms.iswc.ac.cn (L.D.); 2 Academy of Agriculture and Forestry Sciences, Qinghai University, Xining 810016, China; qhxnshuoshen@qhu.edu.cn (S.S.); hychen@qhu.edu.cn (H.C.); zhangyang123@qhu.edu.cn (Y.Z.); yjliuqh@qhu.edu.cn (Y.L.); 3 Key Laboratory of Agricultural Integrated Pest Management of Qinghai Province, Qinghai University, Xining 810016, China; 4 College of Forestry, Northwest A&F University, Xiangyang 712100, China

**Keywords:** flurochloridone, rhizosphere soil, fungal community and composition, next-generation sequencing

## Abstract

The application of herbicides to arable land is still the most effective and accepted method to protect plants from weeds. Extensive use of chemicals in conventional agricultural practices has resulted in continuous and serious environmental pollution. Flurochloridone (FLC) is a monophenyl pyrrolidinone selective herbicide that is commonly used to inhibit weeds that occur during the growth of potatoes. In recent years, research on the toxicity of FLC has gradually increased. However, it is relatively rare to analyze the role of FLC by studying the composition of soil microorganisms. Therefore, we used NGS methods to identify the fungal community structure of the low content soil (LS) and high content soil (HS) samples in this study. Subsequently, we identified the fungal community and composition differences of these two group samples using the statistical analysis. Despite the variances of fungal community and composition across the different samples within the group, the fungal composition of the LS samples and the HS samples. LS samples were predominated by *Ascomycota*, while the HS samples were predominated by *Mortierellomycota* and *Basidiomycota*. The major species in the LS samples were *Plectosphaerella* *cucumerina* and *Trichocladium* *asperum*, whereas the dominant species in the HS samples were *Epicoccum nigrum* and *Cladosporium chasmanthicola*. These results suggested that the LS samples and the HS samples had different rhizosphere soil fungal community and composition changes resulting from implementation of FLC in potato growing areas.

## 1. Introduction

Environmental stresses pose a serious threat to agricultural production globally, and herbicides are among the most widely applied agrochemicals, significantly increasing agricultural productivity and crop yields [1,2,3,4]. Flurochloridone (FLC) is a monophenyl pyrrolidinone selective herbicide that is commonly used to inhibit weeds that occur during the growth of crop plants, such as grains, sunflowers, and potatoes [5,6,7,8,9,10]. FLC can cause bleaching of plant leaves through interference with carotenoid biosynthesis [11]. The target site of FLC in weeds is phytoene desaturase (PDS) [12].

The application of herbicides to arable land is still the most effective and accepted method to protect plants from weeds [2]. Extensive use of chemicals in conventional agricultural practices has resulted in continuous and serious environmental pollution. Because of this, public concerns over the residues of herbicides in the environment and in crops have increased over the last decades [13]. In recent years, research on the toxicity of FLC has gradually increased [5,6,7,8,9,10]. However, it is relatively rare to analyze the role of FLC by studying the composition of soil microorganisms.

The rhizosphere zone, a microclimate of soil that surrounds and is influenced by plant roots, harbors a vast number of soil biota involved in complex biological and ecological processes, and it is considered one of the most dynamic interfaces among ecosystems [14,15]. The importance of the soil microbiome has been recognized for more than a century, and there is a long history of research that describes the microorganisms that inhabit soil, their metabolic capabilities, and their influence on soil fertility [16,17,18]. Most current studies focus on the impact of environmental factors on the composition of bacterial communities [19,20,21]. Here, we will investigate the effects of FLC application on rhizosphere soil fungal community and composition in potato growing areas.

Recent methodological advances have enabled researchers to chart the full extent of soil microbial diversity and to build a more comprehensive understanding of specific microbial controls on soil processes. In particular, the next-generation sequencing (NGS) technique has been successfully applied the rhizosphere soil microbial community and composition, because it can provide more detailed information and in-depth microbial community insights when compared to other molecular biology methods [22,23,24,25,26].

In this study, the NGS technique was used to analyze the role of FLC by studying the composition of rhizosphere soil fungal microorganisms in potato growing areas of Qinghai-Tibet Plateau in China. We systematically compared the differences of the fungal communities in FLC application soil. The fungal microbial compositions were determined by the NGS technique of the internal transcribed spacer (ITS) region. This work is to determine rhizosphere soil fungal community and composition changes resulting from implementation of FLC in potato growing areas. It aims to clarify the issues of its dosage, period of use, method of use and safety, and provide a data basis for the large-scale application of the agent in the Qinghai-Tibet Plateau and even the other potato fields.

## 2. Materials and Methods

### 2.1. Field Trials, Soil Sampling, and Preparation

The soil sampling was conducted in a potato growing areas in Qinghai-Tibet Plateau, Qinghai province. The location was at a longitude of 89°35′–103°04′ E and latitude of 31°39′–39°19′ N. The FLC and other chemicals used in the present study were of analytical grade from Sinopharm Chemical Reagent Co., Ltd. (Beijing, China). The experimental soil used in the study was castanozems. The soil was mixed thoroughly with 750 (low content soil, LS) and 1125 (high content soil, HS) g/hm^2^ FLC. After 1, 3, 6, 9, 12, 15, 18, 21, 24, 27, 30, 33, 36, 39, 42, 45, and 48 days, each sample comprised three biological replicates, and the rhizosphere soil samples were collected, respectively. The roots of each plant were gently shaken to remove the redundant soil, and the remaining soil adhered to the root hairs was collected with a brush and sieved with a 2-mm aperture sieve. The specific method details refer to previous research [27,28]. In total, 108 rhizosphere soil samples were collected to analyze the fungal community and composition by NGS technique sequencing.

### 2.2. Soil Properties Analysis

In order to investigate the changes of physicochemical properties, seven soil physicochemical were analyzed. The soil pH was determined using a calibrated pH meter (PHS-3C, LeiCi) by the microelectrode method [29]. The soil organic matter (OM) content was determined using thermal dichromate oxidation colorimetry [30]. Available potassium was determined in ammonium acetate extracts by flame photometry. Available phosphorus was extracted with 0.025 mol × L^−1^ HCl and 0.03 mol × L^−1^ NH^4^F and measured by a spectrophotometer. Soil TP was determined colorimetrically using the molybdate method. Total nitrogen (TN) content determined by Kjeldahl digestion method.

### 2.3. Next-Generation Sequencing

Total genome DNA from rhizosphere soil samples was extracted using the soil DNA extraction kit following the protocol provided by the manufacturer (MoBio Power Soil DNA extraction kit 12888-50, Guangzhou, China). DNA concentration and purity was monitored on 1% agarose gels. According to the concentration, DNA was diluted to 1 ng/µL using sterile water.

The internal transcribed spacer regions of the ribosomal RNA gene were amplified by PCR using the primers ITS1-1F-F CTTGGTCATTTAGAGGAAGTAA, ITS1-1F-R GCTGCGTTCTTCATCGATGC [31,32]. All PCR reactions were carried out with 15 µL of Phusion^®^ High-Fidelity PCR Master Mix (New England Biolabs, Ipswich, MA, USA); 2 µM of forward and reverse primers, and about 10 ng template DNA. Thermal cycling consisted of initial denaturation at 98 °C for 1 min, followed by 30 cycles of denaturation at 98 °C for 10 s, annealing at 50 °C for 30 s, and elongation at 72 °C for 30 s. Finally, 72 °C for 5 min. The “barcode” is an eight-base sequence unique to each sample. Mix same volume of 1x loading buffer (contained SYB green) with PCR products and operate electrophoresis on 2% agarose gel for detection. PCR products was mixed in equidensity ratios. Then, mixture PCR products was purified with Qiagen Gel Extraction Kit (Qiagen, Dusseldorf, Germany).

The sequencing libraries were generated using TruSeq^®^ DNA PCR-Free Sample Preparation Kit (Illumina, San Diego, CA, USA) following the manufacturer’s recommendations, and index codes were added. The library quality was assessed on the Qubit@ 2.0 Fluorometer (Thermo Scientific, Waltham, MA, USA) and Agilent Bioanalyzer 2100 system. Finally, the library was sequenced on an Illumina NovaSeq platform and 250 bp paired-end reads were generated.

### 2.4. Sequence Analysis Processing

The paired-end reads were assigned to samples based on their unique barcodes and truncated by cutting off the barcode and primer sequence. Paired-end reads were merged using FLASH (V1.2.7, http://ccb.jhu.edu/software/FLASH/, accessed on 22 May 2021) [32], a very fast and accurate analysis tool, which was designed to merge paired-end reads when at least some of the reads overlapped the read generated from the opposite end of the same DNA fragment, and the splicing sequences were called raw tags; quality filtering on the raw tags were performed under specific filtering conditions to obtain the high-quality clean tags according to the QIIME (V1.9.1, http://qiime.org/scripts/split_libraries_fastq.html, accessed on 22 May 2021) quality control process [33,34]. In brief, raw tags were truncated from the first low-quality base site, in a row, with low quality value (default quality threshold ≤19), and the set length (default length value is 3). Next, further filtering out the continuous high quality base length was less than the length of the tags (75% of tags), after obtaining the tags data set. The tags were compared with the reference database (Silva database) using the UCHIME algorithm (http://www.drive5.com/usearch/manual/uchime_algo.html, accessed on 22 May 2021) to detect chimera sequences, and then the chimera sequences were removed. Then the effective tags were finally obtained.

The sequence analyses were performed by Uparse software (Uparse v7.0.1001, http://drive5.com/uparse/, accessed on 22 May 2021) [35]. Sequences with ≥97% similarity were assigned to the same OTUs. A representative sequence for each OTU was screened for further annotation [36]. Alpha diversity was applied in analyzing complexity of species diversity for a sample through six indices: observed-species, Chao1, Shannon, Simpson, ACE, good-coverage. All of the indices in our samples were calculated with QIIME (Version 1.7.0) and displayed with R software (Version 2.15.3). Beta diversity analysis was used to evaluate differences of samples in species complexity, Beta diversity on both weighted and unweighted UniFrac were calculated by QIIME software (Version 1.9.1).

## 3. Results and Discussion

### 3.1. Physicochemical Properties

As shown in Table S1, the TN contents of the LS samples and the HS samples ranged from 1.51 to 1.58 g/kg and 1.40 to 1.47 g/kg, respectively. The AN contents of the LS samples varied from 149.33 to 157.21 g/kg, whereas the HS samples had a lower AN (129.45–140.29 g/kg). The OM contents (26.89–28.48 g/kg) in the LS samples were higher than those in the HS samples (24.09–25.24 g/kg). The results TP, TK, AP, AK content showed no significant differences in the LS samples and the HS samples. The pH values between the LS samples and the HS samples also showed no significant differences. Suggesting that the FLC may affect rhizosphere soil microorganisms by changing the contents of nitrogen and OM. Nitrogen fertilizer input provided soil microorganisms simultaneously with N, and the N requiring components for microbial biomass [37,38,39,40,41].

### 3.2. Phylogenetic Composition and Alpha-Diversity

**Figure 1:**
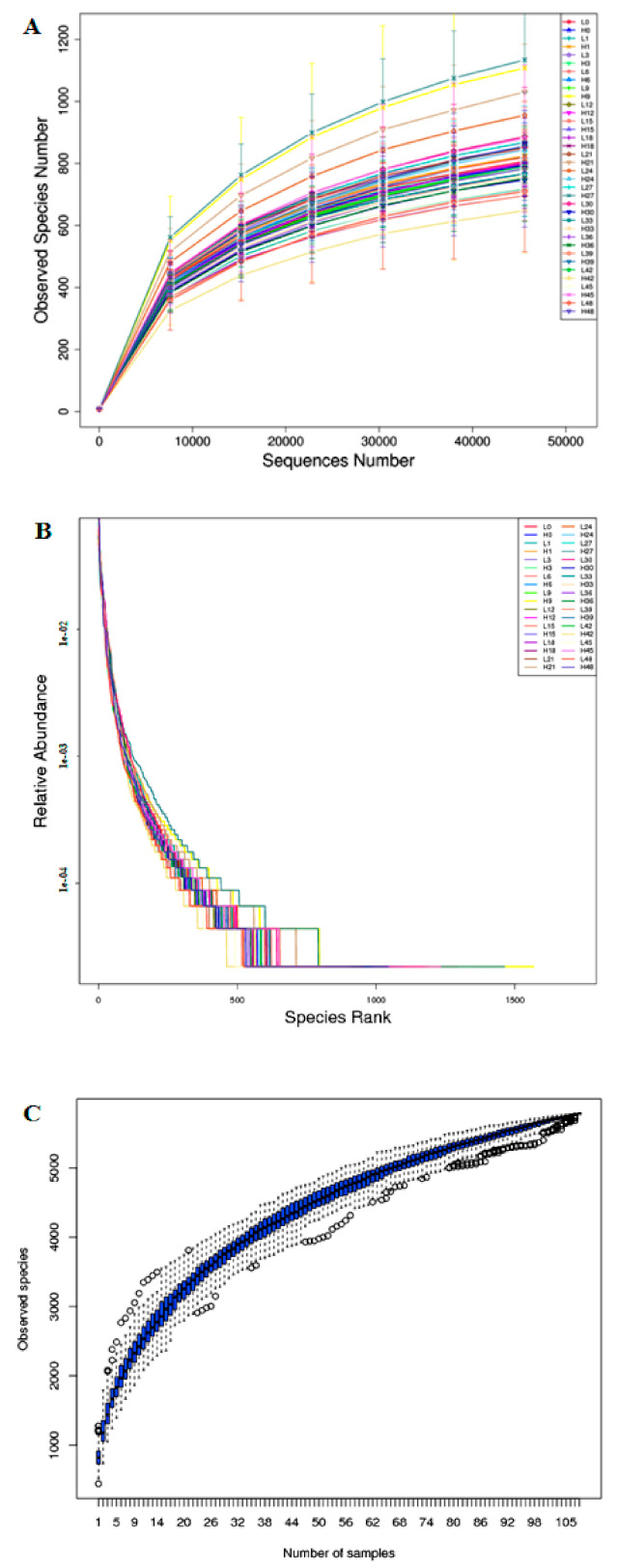
The rarefaction curves (**A**), rank abundance (**B**), and species accumulation curves (**C**) analysis.

**Figure 2:**
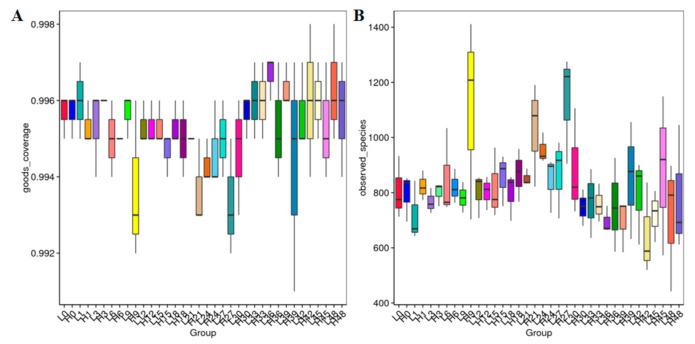
The alpha-diversity index (Observed_species index (**A**) and Shannon index (**B**)) of the LS and HS samples fungal diversity.

An average of 89,010 per samples were obtained from the 108 samples (the 9,641,840 clean reads in total) (Table S2).These samples are based on the rarefaction curve, and the rank abundance analysis by 97% similarity of OTU (Figure 1A,B) shows that our sequencing depth meets the requirements of rhizosphere soil fungal microorganism sequencing and analysis. In addition, the fungal microorganism species accumulation curve shows that our samples are sufficient for OTU testing and can predict the species richness of the samples (Figure 1C). The good coverage value of each sample was >0.99, indicating that the rhizosphere soil fungal community and composition information is sufficient to reveal most fungal communities. The alpha-diversity of the results suggesting that the LS sample fungal diversity was higher than that of the HS samples (Figure 2 and Table S3).

### 3.3. Rhizosphere Soil Fungal Community and Composition

Sequencing the ITS gene of all the soil samples showed that the soil fungal communities in all samples covered 14 phyla, 42 classes, 99 families, 204 families, 394 genera, and 405 species.

**Figure 3:**
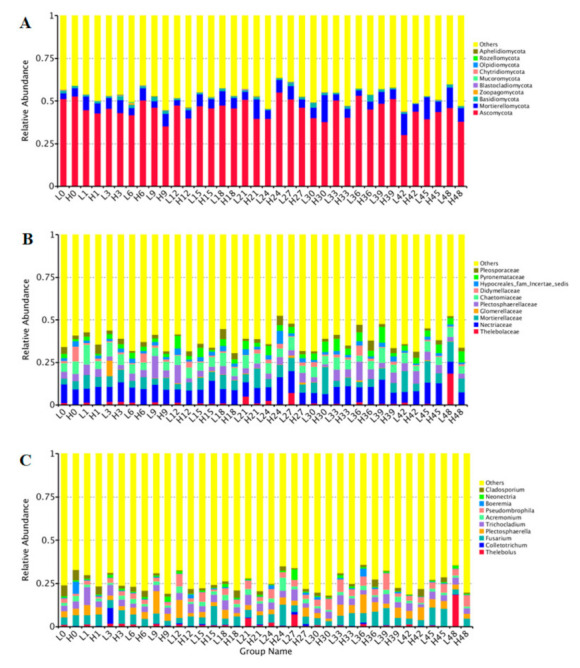
Relative abundance of bacterial community compositions at phylum (**A**), family (**B**), or genus (**C**) levels.

At the phylum level (Figure 3A), *Ascomycota* was the most important phylum in LS and HS samples. The relative abundance of *Ascomycota* in the LS samples was higher than that in the HS samples. In contrast, the relative abundance of *Mortierellomycota* and *Basidiomycota* in the LS samples was lower than that in the HS samples.

*Ascomycota* plays an important role in the degradation of rhizosphere soil organic matter. It is speculated that the decrease in relative abundance of *Ascomycota* may be related to the decline of soil organic matter and soil fertility [42,43].

At the family level (Figure 3B), the more importantrhizosphere soil fungal communities in the LS samples and the HS samples belonged to the top 10 families (*Thelebolaceae*, *Nectriaceae*, *Mortierellaceae*, *Glomerellaceae*, *Plectosphaerellaceae*, *Chaetomiaceae*, *Didymellaceae*, *Hypocreales_fam_Incertae_sedis*, *Pyronemataceae,* and *Pleosporaceae*). Overall, *Thelebolaceae* was the most abundant family in both LS and HS samples. The proportions of *Thelebolaceae* in the LS samples were higher than that in the HS samples.

At the genera level (Figure 3C), *Thelebolus* was the most abundant in all the LS samples and the HS samples. The other genus with high abundance, included *Colletotrichum*, *Fusarium*, *Plectosphaerella*, *Trichocladium*, *Acremonium*, *Pseudombrophila*, *Boeremia*, *Neonectria,* and *Cladosporium*, respectively. In general, *Thelebolus* was more abundant in the LS samples than that in the HS samples, whereas *Colletotrichum* and *Fusarium* in the LS samples was less than in HS samples. At the genus level, *Cadophora* and *Tetracladium* were the dominant microorganisms among the plant mycobiota. Although their OTU abundances were different, both *Cadophora* and *Tetracladium* belong to *Ascomycota*, which is considered the most likely endophytic taxon to colonize plants [44]. *Ascomycota* is widespread in soil and plants, such as forage, flowers, and crops [45,46,47].

The top 10 species in both LS and HS samples included *Thelebolus microsporus*, *Plectosphaerella cucumerina*, *Trichocladium asperum*, *Pseudombrophila hepatica*, *Boeremia exigua*, *Epicoccum_nigrum*, *Cladosporium chasmanthicola*, *Alternaria alternata*, *Metarhizium robertsii* and *Gibberella pulicaris* (Table S4). The relative abundance of *Thelebolus microsporus* in the soil LS samples was much higher than that in the HS samples. The relative abundance of *Plectosphaerella cucumerina* and *Trichocladium asperum* in the soil LS samples were also higher than that in the HS samples, while the relative abundance of *Epicoccum_nigrum* and *Cladosporium chasmanthicola* in the soil LS samples were also lower than that in the HS samples.

### 3.4. Comparison between LS and HS Samples

**Figure 4:**
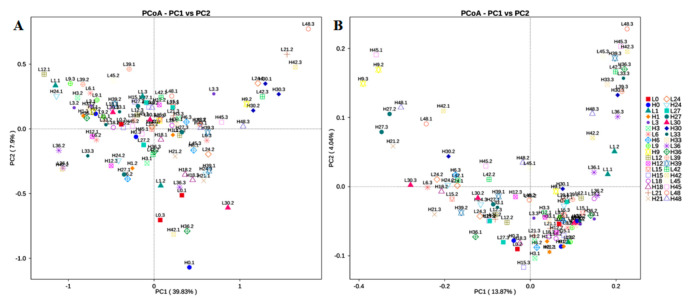
The UniFrac unweighted (**A**) and the weighted (**B**) principal coordinate analysis (PCoA) scores plot based on principal components PC1 and PC2.

Based on weighted and unweighted UniFrac distances, the PcoA showed the separation of the soil fungi communities between the LS samples and the HS samples (Figure 4A,B). By the weighted UniFrac distances, the values of cumulative percentage variance of species in the first (PC1) and the second (PC2) axes were 39.83% and 7.90%, respectively. In addition, according to the unweighted UniFrac distance, the first (PC1) and the second (PC2) axes accounted for 18.37% and 4.04% of the variance in the soil fungi communities of samples, respectively.

**Figure 5:**
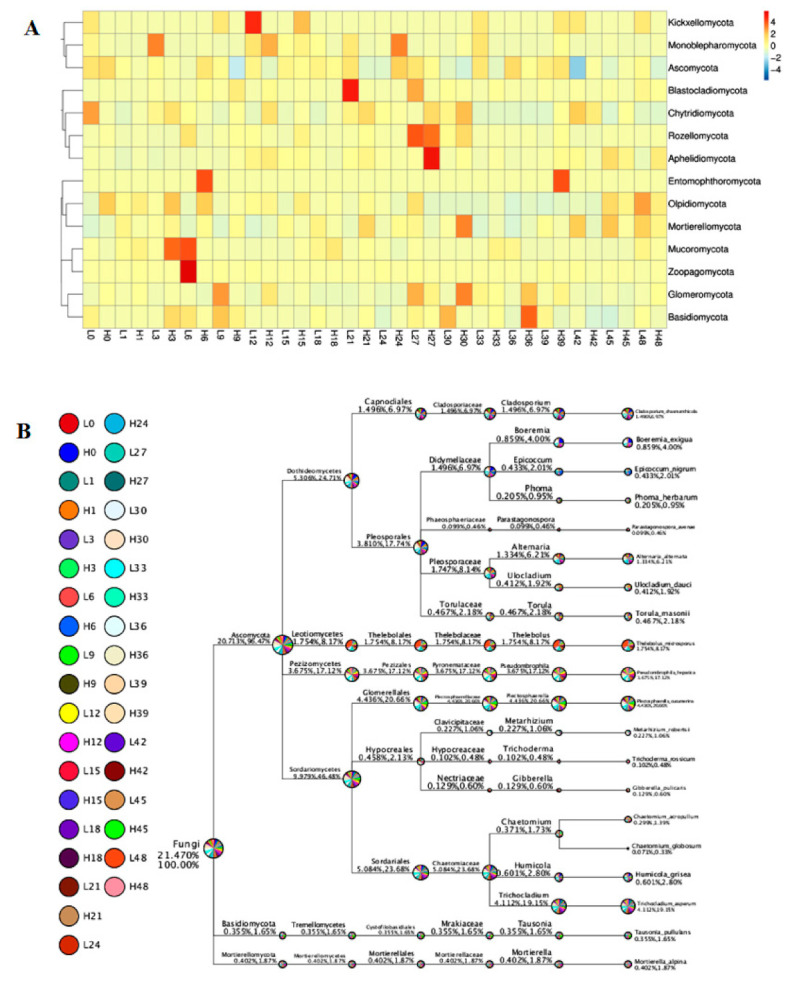
The LS samples and the HS samples fungal community heatmap analysis (**A**) and similarity tree (**B**).

The differences and similarities of soil fungal community structures among samples were analyzed and compared using heat map analysis (Figure 5). As shown in Figure 5, the proportion of *Thelebolaceae* were more abundant in the soil LS samples, whereas the soil fungal communities, such as *Chytridiomycota*, *Aphelidiomycota*, *Entomophthoromycota*, and *Basidiomycota,* were more abundant in the soil HS samples.

**Figure 6:**
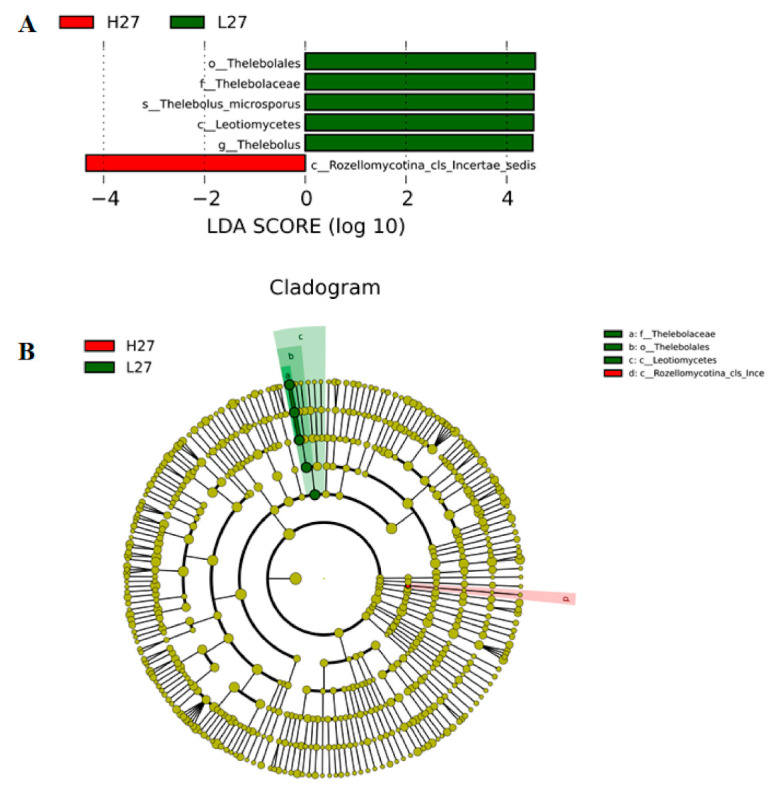
The LEfSe analyses between the LS (L27) sample and the HS (H27) sample. (**A**) Histogram of the results of the LS (L27) sample and the HS (H27) sample. (**B**) Cladogram representing the abundance of the LS (L27) sample and the HS (H27) sample. Green: L27; Red: H27.

The Linear Discriminant Analysis Effect Size (LEfSe) analysis was used to determine the significant difference of the fungal communities between the LS (L27) samples and the HS (H27) samples (Figure 6A,B). The relative abundances of the *Thelebolales Thelebolaceae* was significantly higher in the LS (L27) samples, while the *Rozellomycotina_cls_Incertae_sedis* was higher in the HS (H27) samples.

The rhizosphere soil fungal community and composition take on the role of soil decomposers. Different types and numbers of fungal communities play an important role in the process of material and energy cycles. They can convert complex and refractory organic matter, which is transformed into nutrient elements that plants can use [48,49,50].

## 4. Conclusions

We used the NGS technique to identify the rhizosphere soil fungal community and composition in the LS samples and the HS samples, and compared their fungal microbial and physicochemical properties. The 14 phyla, 42 classes, 99 orders, 204 families, 394 genera, and 405 species were identified in all of the rhizosphere soil samples. Despite the variances of soil fungal communities and compositions across the different samples, the fungal community, and composition of the LS samples and the HS samples, the LS samples were predominated by *Ascomycota*, while the HS samples were predominated by *Mortierellomycota* and *Basidiomycota*. The major species in the LS samples were *Plectosphaerella cucumerina* and *Trichocladium asperum*, whereas the dominant species in the HS samples were *Epicoccum nigrum* and *Cladosporium chasmanthicola*. These results provided a basic understanding of the fungal community and composition and the significant difference of fungal profiles in the LS samples and the HS samplesafter FLC application. However, the roles of these fungal communities in the rhizosphere soil in potato growing areasof the Qinghai-Tibet Plateau are still unknown, further research is needed to investigate the roles of these species in the rhizosphere soil.

## Data Availability

Data is contained within the article or supplementary material. It is also available from the author Wei Li (Liweilwbabylw@qhu.edu.cn).

## Supplementary Materials

The following are available online at https://www.mdpi.com/article/10.3390/jof7060420/s1. Table S1. The pysicochemical properties of the LS samples and the HS samples; Table S2. Total number of reads, read length and quality control (QC) of the LS samples and the HS samples; Table S3. The alpha-diversity index and good’s coverage for the soil fungal community and composition information per sample; Table S4. The abundance of the top 10 species in the LS samples and the HS samples.
